# Medico-Chirurgical Transactions. Vol. XXIII

**Published:** 1841-01-01

**Authors:** 


### Medico-Chirurgical Review,

### No. LXVII.

[No. 27 of a Decennial Series.]

OCTOBER 1, 1840, TO JANUARY 1, 1841.

Medico-chirurgical Transactions, Published by the Royal Medical and Chirurgical Society of London. Vol. XXIII. London : Longman and Co. 1840.

This admirable Society is increasing in extent and honour, and, certainly, not retrograding in usefulness. All other societies, in this Kingdom, may own without a blush its superiority, and its European reputation is of a high order. To be a member of it, is courted as a distinction, and to be a contributor to its Transactions is a guarantee of merit.

The present volume of those Transactions is of a character very similar to that of its predecessors. The same variety, the same preponderating practical tone which distinguished them, pervade it, and render the task of disseminating its contents among the less opulent members of the profession and those whose lot has placed them in our Colonies or on the ocean, one of equal pleasure and profit. And the members of the Society must themselves feel gratification at every thing which widens the sphere of their utility, and makes them more known to the world.

The contents of the work are as follows:—

1. Case of strangulated hernia, in which the bowel was ruptured by the patient in his efforts to reduce it; by Benjamin Travers.—2. Observations on the blood-corpuscules and pus-globules in certain animals; by George Gulliver.—3. On white spots on the surface of the heart, and on the frequency of pericarditis ; by James Paget.—4. Remarks on emphysema of the lungs ; by George Budd, M.D.—5. On a remarkable effect on the human gums, produced by the absorption of lead ; by Henry Burton, M.D.—6. A case of disease in the posterior columns of the spinal cord ; by Edward Stanley.—7. On the arrangement of the intermediate vessels on surfaces secreting pus, with a note regarding the vascularity of inter-articular cartilages ; by Robert Liston.—8. Remarks on the diagnosis of foreign bodies in the larynx; by Cæsar H. Hawkins.—9. History of a case in which the operation of tracheotomy was performed, with observations; by Benjamin Travers.—10. Second Memoir on some principles of the pathology of the nervous system ; by Marshall Hall, M.D.—11. Third Memoir on the same subject ; by the same author.—12. On the presence of sulphur in cystic oxyde, and an account of a cystic oxyde calculus; by Henry Bence Jones.—13. Case of large osseous tumour of the uterus; by James M. Arnott.—14. On the rapid organization of lymph in cachexia; by John Dalrymple.—15. A case of recovery from cut throat, in which both the larynx and pharynx were extensively opened; by R. A. Stafford.—16. On the structure of the human placenta, and its connexion with the uterus; by William Bloxam.—17. Termination of the case of William Chandler, afflicted with dry gangrene, of which an account was published in the twenty-second volume of the Transactions; by S. Solly.—18. Observations on injuries of joints, and their treatment; by Rutherford Alcock.—19. On aneurisms, and especially spontaneous varicose aneurisms of the ascending aorta, and sinuses of Valsalva, with cases; by John Thurnam.—20. Case of a rare species of hydatid, the echinococcus hominis, found in the human liver; by T. B. Curling.—21. Observations on the mode of union of fractured bones; by R. H. Meade.—22. Case of aneurism of the arteria innominata, in which the carotid and subclavian arteries were tied; by W. Wickham.—23. Case of tumour in the pelvis, impeding parturition; by J. C. W. Lever.

We shall take the Papers as they come, and notice them seriatim.

### I. A Case of Strangulated Hernia, in which the Bowel was ruptured by the Patient in his efforts to reduce it. By Benjamin Travers, Esq. F.R.S.

Mr. Travers is an old and valued contributor. The succeeding case is very interesting.

At 3, a.m. Oct. 29, 1839, Mr. T. was summoned to see a young gentleman, aged 20, supposed to be suffering from strangulated hernia.

Mr. T. found him lying on his back, with a countenance expressive of great suffering and anxiety, his surface pale and chilled, eyes and lips livid, legs drawn upwards, pulse at the wrist imperceptible, and the heart's action rapid and indistinct. He had vomited several times, but had passed a motion at four in the afternoon of the preceding day. The scrotum was enlarged to the size of a young child's head, and discoloured of a gangrenous hue; the skin distended as if about to burst. The hypogastrium was swollen and tense, the pain acute, diffused and much increased on pressure.

It appeared that he had recently laboured under gonorrhœa and swelled testicle on the right side, and that he had been the subject of hernia on the same side, from his birth; that he had not worn a truss since his childhood, but was in the habit of lying down and replacing the protruded bowel with his hands, when inconveniently distended in the sac. At six o'clock of the preceding evening the volume of the tumor became so increased, as to oppose a resistance to its return, which the young man could not overcome. In addition to continued violent manipulation, he compressed it forcibly between his hands and thighs, and as if reckless from ill success, actually made a section of the integument with a razor, transverse to the chord. At midnight, he sent for his father, and told him he had burst his testicle, or that something had given way, and if unassisted, he must die. The bulk of the swelling had gone on increasing, from this time, to the period at which Mr. Travers visited him.

Mr. Travers gave him some warm brandy and water to drink, which restored a very slender pulse, and then made an incision in the tract of the spermatic chord, towards the fundus of the tumor. The subcutaneous cellular membrane was throughout infiltrated with a dark-coloured fluid, emitting a feculent odour, and freely oozing from the section. The subjacent cremaster tunic was then divided, and the collapsed sac, forming an enormous pouch, divided, and incised on a director: the testis lay exposed and somewhat swollen in its lower part, and a flaccid fold of bowel occupied the mouth of the sac, which on handling the parts, slipped into the belly. The finger passed freely through the dilated canal and upper orifice, round the epigastric vessels, into the abdominal cavity. There was nothing like stricture to be perceived at any part. A single stitch was placed in the edges of the sac, which was condensed on the posterior part, and of a loose cellular texture on the anterior and lateral parts. The divided integuments were also connected by a central stitch, over a dossil of lint; a free incision carried through the raphé scroti posteriorly, exhibited a similar loaded state of the cellular membrane behind, as in front, and extending far beyond the mesial line.

This completed the operation: the fluid had escaped in such quantity as to form a pool in the bed. During the operation the patient threw up some frothy bilious fluid, and very soon afterwards had two pretty copious stools of semi-fluid consistence, and precisely of the same colour as the effused intestinal fluid. Temporary relief was obtained, but at seven p.m. three hours after the operation, he died.

*Dissection, ten hours after death.*—The abdominal muscles were rigid, and a vast quantity of offensive flatus escaped on making the ordinary section, and through the scrotal wound. On tracing the small gut, a portion of the lower third of the ileum, equal to a hand's breadth in extent, presented the appearances of recent strangulation, accompanied with laceration, and extravasation of blood between the peritoneal and muscular coats. The fold was collapsed, of a claret colour, bounded by a faint ash-coloured streak at either end, and presenting three or four insulated grey spots of incipient gangrene. An irregular aperture, three-fourths of an inch in length, was found adjacent to the mesenteric attachment, and parallel to the axis of the bowel; and immediately contiguous to this wound, the serous membrane was detached from that beneath it to the extent of an inch, so as to exhibit the circular fibres of the muscular coat, as if dissected. Minute clots of blood were lying in this space, and some extravasation had taken place between the layers of the corresponding mesentery. The neighbouring small intestine was at several points inflamed, and congested even to extravasation beneath the peritoneal investment; liver reduced as if shrunk in bulk; its surface of a dark green hue, and slightly roughened. On section, it was tougher and paler than this tissue ordinarily is in youth and health.

In some excellent observations on this case, Mr. Travers remarks that it was an instance of "*hernie par engouement,*" the strangulation having resulted from the loaded state of the intestine—an accident to which all those who wear no truss are of necessity liable. The great violence employed accounts for the rupture of the gut, though not previously diseased.

Mr. Travers alludes to the circumstances under which rupture of the gastro intestinal tube may occur.

"I have known the intestine ruptured in the taxis, but it was ascertained that the stricture included two-thirds only of the cylinder, which was sloughy and separating by ulceration.

A man received a violent blow from a hammer on the pad of his truss, while stooping, by which the bowel was ruptured. This event was favoured by position, but could not have happened had the truss been efficient.

From compression, as by a wheel passing over the body, or pinning it against a wall, from the kick of a horse, from a fall across a beam, from running violently against a post in the dark, and similar causes, I have known the bowel ruptured; sometimes in more places than one, and sometimes complicated with lesion of the liver, spleen, or kidney, without breach of the walls of the abdomen. Distorted muscular action is sufficient to produce rupture of the stomach in a loaded state, as I witnessed many years ago in the case of a tumbling boy, who a few minutes before had been partaking freely of apples and gin, which were effused.

I never knew a case of ruptured intestine from vomiting or muscular action, unless ulcer had previously existed, by which the internal tunics were destroyed; in this case the peritoneum covering the aperture is probably burst, for the symptoms of effusion commence almost simultaneously with the act of vomiting. This act, I may observe, is abridged and impeded after rupture of the canal at any part; it is half vomiting and half expectoration, as if the muscles had lost their fulcrum, which is in fact the case; the fluid rises into the fauces, and is with a convulsive effort spit our of the mouth." 8.

Mr. Travers' account of the symptoms is graphic and concise. They are death-like, he says, from the moment of the injury. The mind is clear but depressed, as if overwhelmed by the irreparable nature of the injury. The countenance is pale and the features liny and drawn. The pulse is not immediately affected, but soon becomes quick, feeble, and irregular in its measure, intermitting, thready, and then no longer to be felt. The surface chills, but remains dry; there is a painful sense of dryness of the mouth and fauces, and frequent efforts to vomit in the way before described. Pain, which commences at variable periods, but is never long delayed, is acute, unremitting, extending over the whole abdominal region, which becomes tense and will not bear the slightest pressure. This produces great anxiety and restlessness, and frequent appeals for relief, and next for death. The peritoneal surface is reddened, but there is seldom any effusion of membranous or massive lymph agglutinating parts; only small deposits in tags and shreds roughening the surface, although the period of survival varies from twelve to six-and-thirty hours: the state of the canal perhaps determines this variation.

He naturally refers the prostration, &c. rather to the noxious effect of the effused intestinal air and contents on the nervous and absorbent tissue of the peritoneum. He has seen *pain* so little marked, as to lead to a doubt in diagnosis. With regard to treatment he has nothing to suggest, unless it be an absolute negation of food and diluent.

Mr. Travers next relates two cases with the view of urging his objections against the plan of leaving the sac unopened in the operation for hernia.

In the *first* case that proceeding was perfectly successful.

The *second*, was one of femoral hernia, in a man aged 55. The symptoms of strangulation had existed for three days. The usual means of relief having failed, the operation was done without delay. There was found, beneath a suppurating lymphatic gland, a small and very tense sac. The fibres of the crural arch were divided upon the point of the finger; but it was found necessary to carry the probe-pointed bistoury under the arched fibres of the fascia transversalis, in order to liberate the contents of the sac, which returned with a gurgling noise into the belly. The return of the gut, and collapse of the sac, were perfect and satisfactory; the latter was in consequance not opened. The patient was imperfectly relieved; vomiting continued at intervals throughout the rest of the day and night, although several scanty, dark, and scybalous stools were obtained by injection, and small doses of aperient salts.

Evacuations continued, but on the fourth day bilious vomiting recurred, followed by abdominal pain, and constitutional depression. On the eighth day he died.

*Dissection twelve hours after death.*—Stomach distended ; small intestines moderately full ; peritoneum presented some red specks at the angles of contact of the intestinal folds. A portion of the tube of the ileum was disorganised, being an ash-coloured rag ; but an adhesion at the mouth of the sac included this piece, so that it was not seen on opening the abdomen, nor until the adhesion gave way, when a quantity of pultaceous feculent matter passed into the pelvis. Another fold of intestine adhered to the sound side of this, and supported it against the ring. A portion equal to one-third of the canal of the strictured gut was sound, and freely admitted the passage of a bougie, and by this route the feculent matter had passed from the upper to the lower part of the tract. On the mouth of the sac was deposited a lip or elevated border of lymph, corresponding to the line of separation of the slough from the living edge, so that no effusion into the belly could have taken place.

Mr. Travers observes that an artificial anus would probably have saved the man. But not only, he adds, was no benefit gained, but the separating process was retarded by the integrity of the peritoneal sac. Had this been opened by the ordinary incision at the time of operation, both the sloughing and the adhesive process would have been so much accelerated, as to have established the artificial anus in time for the patient's relief, and ultimately, perhaps, the continuity of the canal.

The following are Mr. Travers' opinions on the operation we have been discussing.

"I believe that the advantage is altogether hypothetical which is supposed to accrue from preserving the sac entire, in cases where the gut is simply paralysed, and unable to resume its function, the ordinary cause of failure of the operation for hernia ; and that the practice is decidedly disadvantageous in all cases where inflammation is of such standing as to have endangered the continuity of the canal; for of such cases, doubtful as is the alternative, the only chance of recovery is in the speedy relief of the symptoms by artificial anus. The incision of the sac establishes at once a free fistulous opening into the peritoneal cavity, and identifies it with the external wound, which materially quickens the separating, and strengthens the fastening process; the spoiled bowel being left *in situ* at the mouth of the sac." 16.

"But not to insist upon the numerous cases in which the seat of stricture or the existence of adhesions does not permit us to leave the sac entire, the practice is objectionable, on the ground that where the stricture is of such firmness as to require the aid of the knife, we can never know the actual state of the bowel, with which it is the paramount duty of the surgeon to make himself acquainted, that he may regulate his proceedings accordingly." 17.

We fancy there can be no doubt of the occasional success, indeed propriety, of leaving the sac unopened. But the uncertainty that attends it, the possibility, however slight, of leaving a source of mischief uncontrolled, and the questionable aggravation of risk which opening the sac occasions, will always tend to limit the employment and patrons of the measure.

The next observations are, it seems to us, extremely just.

"I have adverted to the paralysed condition of the strangulated bowel, as the ordinary cause of death after the operation for hernia. This opinion, which I published near thirty years ago, has been fully confirmed by my larger experience; yet it is not, I believe, generally entertained. Death is ascribed to the inflammation of the general cavity, where gangrene of the gut has not supervened upon its replacement: but in how few cases does inflammation prove intractable, when the operation is followed by full evacuations, demonstrating the re-establishment of the canal. I have repeatedly seen patients, after operation, in whom the symptoms of inflammation were feebly, if at all, indicated, and have inspected many 'post mortem,' in whom no agglutination interfering with the function of the bowel had taken place, or other explanation of the continued obstruction showed itself, than the utter atony of the congested bowel, which had been replaced; marked by its precise interposition to the flatulent portion of the tube above, and the collapsed piece below." 18.

We believe that this opinion is now obtaining a general assent among all well informed surgeons,

Mr. Travers speaks favourably, and surgeons will generally, we think, echo his opinion, of the effects of calomel both in cases of inflammation of the bowels, and of that paralysed state of it which has been referred to. In the latter instance, it stimulates the liver, and promotes that downward flow of bile, which being the most natural is, perhaps, also the best stimulus to the bowel.

### II. OBSERVATIONS ON THE BLOOD-CORPUSCLES AND PUS-GLOBULES IN CERTAIN ANIMALS. By GEORGE GULLIVER, Esq. &c. &c.

Since the time of John Hunter, it has been supposed that the globules of pus are merely the red particles of blood, deprived of their colouring matter, and modified in form and size by the inflammatory process. But Müller and Dr. Güterbock have doubted this, and Mr. Gulliver has lately been setting himself to work to determine it. He found this, however, no easy matter, and the following admission which winds up some experiments on the pus of the dromedary, the paco, the guanaco, or wild lama, and the vicugna (all mammals with elliptical blood disks, and therefore, by reason of this peculiarity, well calculated, apparently, to determine the connexion between *them* and the pus-globules), will shew that the question is still to be decided.

"Without asserting the impossibility of a transformation of the blood-disk into the globule of pus, it can hardly be supposed that any such change took place in the experiments recorded in this paper. This question, however, appears to me to be one of more difficult solution by mere microscopic observation than would be supposed by any one who had not specially examined the subject; for the blood-corpuscules are so singularly susceptible of modifications in form, size and general characters from very slight agency, that examples might readily be shown of their approximation in appearance to the globules of pus. The action of water on the blood-disks of the mammalia, as well as of the lower vertebrate animals, has been well known since the time of Hewson.

Now, however, that so much attention is devoted to the constitution of the healthy as well as morbid animal fluids, the relation, if any, between the blood corpuscles and the particles of the secretions will probably be soon finally determined. From my observations it appears that the blood-disks of the goat are by no means the smallest among the mammalia, as had been previously supposed, but that the blood-corpuscles of the napu musk deer, and probably of its congeners, are so singularly minute that their average diameter is between $\frac{1}{14000}$th and $\frac{1}{12000}$th of an inch. It would, therefore, be interesting to examine the pus of an animal of this genus. In the meantime it may be mentioned that I found in the blood of the musk deer several large white spherical bodies, similar to those observable in the blood of other animals, and that the lymph globules did not differ in magnitude from those of mammals with large blood-corpuscles."

### III. On White Spots on the Surface of the Heart, and on the frequency of Pericarditis. By James Paget, Esq.

Mr. Paget observes that these spots occur most frequently on the anterior surface of the right ventricle, and are often found tracking out the course of the trunks of its coronary vessels. They are rather more rarely seen on the posterior surface of the right ventricle, and on the right auricle; more rarely still on the left ventricle, and most rarely of all on the left auricle.

With these spots there almost constantly coincides some adhesion, by organised lymph, between adjacent parts of the pericardial membrane, which leads Mr. Paget to regard them as, in all cases, the results of pericarditis.

The adhesions generally consist of slender threads passing across the furrow between the aorta and vena cava superior, or between the aorta and pulmonary artery at some little distance from their connexion with the heart. In other cases, they are attached by one extremity to either of these vessels, and by the other to the opposite surface of the pericardium; and more rarely, though more distinctly, a band of adhesion passes from one of the spots or from some adjacent part of the heart to the opposite surface. Lastly, there may be found only the indications of adhesions that had once existed, in the form of small pearly granules on the surface of the aorta or cava, and on the corresponding surface of the pericardium. They sometimes require so careful an examination to find them that Mr. Paget feels no surprise at their being overlooked.

He finds than in 40 cases in which there were white spots on the heart, 35 have presented anormal adhesions, or their remains. In 5 cases only were the adhesions absent, and in 4 cases only an adhesion was found where there were no spots. In the 35 cases with adhesions, they were situated 23 times between the aorta and some other vessel, and 19 times between one of the great vessels and the opposite surface of the pericardium. In four cases he has found a band of adhesion passing from the surface of a spot to the pericardium opposite to it, and Dr. Budd has twice seen a similar formation. In many cases also there is a distinct roughness or radiated puckering, like a superficial cicatrix, on the pericardium opposite the spots. In these it is probable that the spots are indications of the pre-existence of adhesions; but in all other cases Mr. Paget would regard them as the effects of local and defined inflammation, which has been prevented from producing adhesion by the fluid simultaneously effused separating the serous surfaces, and permitting part of the lymph to sink down to the great vessels, while the rest remained on the surface and in the cellular tissue of the inflamed part.

Mr. Paget thinks the question—whether these spots be seated in or on the pericardial membrane of the heart, one of little moment, this depending on the depth and degree to which the cellular or adipose tissue round the heart is inflamed. He believes that the reason of the frequency of the adhesions about the great vessels is, that in comparison with the walls of the heart, *they* are fixed and motionless, and present every facility for the adhesion and organization of the lymph that is effused, and which either gravitates to them in the recumbent position of the body, or is impelled thither by the currents which the action of the heart excites in the fluid around it.

The white spots have been said to occur "in half, or more than half, of those who are above the age of childhood. M. Bizot found them 45 times in 156 subjects; viz., in 31 out of 72 men, and in 14 out of 84 women; and I have myself found them in 45 cases out of 110; viz. in 32 out of 66 males, and in 13 out of 44 females." Mr. Paget thinks that both the white spots and the adhesions cannot but be regarded as the effects of inflammation of some part of the pericardium. This he lays down as an axiom.

"Including then the white spots among the effects of pericarditis, I find that of 110 cases which I have lately examined at St. Bartholomew's Hospital, 58 have presented signs of having suffered at some time from that disease. Among these, 40 out of 66 males, and 18 out of 44 females, were thus affected; and with respect to their ages, the morbid appearances were found in 5 out of 14 below twenty; in 25 out of 53 between the ages of twenty and forty; and in 28 out of 43 above forty.

Of these 58 cases of pericarditis, 49 were slight cases, marked by white spots and adhesions, or by effusion of small quantities of lymph; and 9 were severe with complete adhesion, or with abundant recent effusion.

The subsequent effects of the slight cases of pericarditis are not appreciable. In none of them was there any disease of the heart, but such as was sufficiently accounted for by some other coincident affection, as disease of the valves, &c. In three cases of complete and close adhesion of the pericardial surfaces also, in which there was no coincident disease of the valves, the patients were engaged in active work, and died of affections over which the state of the heart had no evident influence. In two other cases of complete adhesion, the valves were diseased, and both these proved fatal; one in three years, and the other in a year after the first attack, which occurred in the course of rheumatism." 35.

He has not been able to ascertain the circumstances under which these slight affections of the pericardium have happened. He has several times seen such, as an accident of typhus fever, and he thinks it may exist in the course of many diseases. Of the 66 males examined, 24 were known drunkards; and of these 20, had had pericarditis; a proportion sufficient to prove that intemperance and its consequences are among the most powerful excitants of this disease.

This really appears to us to be a paper very creditable to its author. We rather think he is attached to the School of St. Bartholomew's Hospital. We anticipate further communications of value from the same hand. For our own parts we feel quite convinced of the justice of Mr. Paget's conclusions. They give statistical corroboration to an opinion which the analogy of the pleura would render the most probable.

### IV.—Remarks on Emphysema of the Lungs. By George Budd, M.D. &c. &c.

It is one of the chief objects of Dr. Budd's paper to show that want of elasticity in the lung—in other words, absence of its natural tendency to collapse,—is the cause of many of the other anatomical characters of emphysema, and of most of the symptoms by which this affection is recognized. He observes, that the *elasticity* of the lung is one of the most efficient, if not the most efficient agent in expiration, the lung collapsing by virtue of it, and the parietes of the chest subsiding.

"One of the first effects of this condition is, that the lungs, and with them the parietes of the chest, do not collapse as they should do in expiration; the powerful muscles of inspiration are continually acting to elevate the ribs and dilate the chest, and have not their natural antagonist. The chest becomes, in consequence, permanently dilated; often beyond the limit attained in the most ample natural inspiration. It is the permanent elevation of the ribs that gives to the chest the cylindrical form, and, by raising in turn the shoulder-blades and collar-bones, that produces the high shoulders of asthmatic persons.

When this conformation of the chest is attained, its capacity cannot be much further increased by the action of the muscles, which raise the ribs. This circumstance gives a peculiar character to the breathing of persons affected with emphysema; the ribs being permanently raised by the dilatation of the chest, the increased capacity of that cavity which takes place in inspiration is mainly effected by the diaphragm, and the respiration is abdominal. It is owing to this circumstance that the erect posture is more necessary to asthmatics than to persons affected with pleurisy or pneumonia, in whom the respiration is of equal, or even greater, frequency; and that dyspepsia, by causing flatulence and distension of the stomach, and so opposing the descent of the diaphragm, is so often the cause of a fit. The attack of the paroxysm in the night—a peculiar feature of asthma—seems to result, not from the state of sleep, but from the horizontal posture, which causes impediment to the descent of the diaphragm. The abdominal character of the breathing is still further increased by the circumstance that the portion of lung in contact with the diaphragm is not so subject to emphysema as others. This character of the breathing is very conspicuous in horses affected with emphysema, on account of the shortness of their flanks, and is well known to horse-dealers as a sign of broken-wind." 41.

Another circumstance, is the almost fixture of the ribs, which remain comparatively motionless amongst all the dyspnœa.

The *cough* of asthma is peculiar. We may observe that the parietes of the chest are little affected by it, and that it is short and interrupted; a circumstance the more distressing because the catarrh, to which persons affected with emphysema are habitually subject, is attended with a copious secretion from the bronchial membrane. The efforts of cough being ineffectual, and the irritation of the mucus remaining, the cough repeats itself in fits. Thus, in this distressing complaint, not only is less air than natural admitted to the internal surface of the lungs, but that surface is also sheathed from its action by a copious secretion which the cough is inadequate to detach. Catarrh, then, is the great enemy of the asthmatic, and change of climate the great remedy.

When the emphysema is less considerable, or only partial, of course its influence will be proportionably less. The emphysematous portion acting less, the healthy must act more. The air entering the former imperfectly produces a feeble respiratory murmur. Less blood too passes to the morbid lung. Dr. Budd insists on this, quoting two cases of Laennec's, and one of his own in illustration. We may introduce the latter.

In the winter of 1837, a man was admitted into the Dreadnought, affected with general emphysema of the lungs and pulmonary catarrh. He died in a state of asphyxia soon after admission.

The lungs were found extremely dry and pale; there was dark blood in the large veins of the lungs; but, except from these, scarcely a drop of blood escaped when free incisions were made in all parts of the lung. There was no pneumonia, but the small bronchial tubes contained yellow puriform mucus.

He adds:—

"These cases are very striking; for what can be more remarkable than to find paleness, dryness, and absence of congestion in the lungs of persons who have died in a state of asphyxia; the well-known and most marked effect of that condition being the greatest possible congestion of the lungs! The conclusion is, therefore, peremptory, that during life the natural vascularity of the lungs, at least as regards the pulmonary artery, was much diminished. This restriction is necessary; since, in the cases referred to, the bronchial membrane was red and turgid. The coincidence of the pale, aneuric condition of the pulmonary tissue, with the congested state of the mucous membrane of the bronchial tubes, in the same lung, is worthy of observation, as showing an essential difference between bronchitis and pneumonia—a difference which has its origin in the different purpose and distribution of the bronchial and pulmonary arteries." 46.

One effect of this condition of the lung is imperfect arterialization of the blood, and, consequently, diminution of animal heat.

Another consequence of this diminution of the capillary system of the pulmonary artery, is obstruction to the circulation through it: whence arise dilatation of the right cavities of the heart, and the tendency to general œdema, which is so frequently met with in emphysematous persons.

Dr. Budd alludes again to broken wind in horses, and shews, from twenty dissections, its dependence on emphysema of the lungs.

Mr. Jackson found that, of twenty-eight persons affected with emphysema of the lungs, he found that eighteen were the offspring of parents (father or mother) affected with the same disease, and that several of these had died in its course. In some instances, the brothers and sisters of these persons were also emphysematous. On the other hand, of fifty persons not affected with emphysema of the lungs, three only were the offspring of emphysematous parents: whence it follows that emphysema is very frequently an hereditary disease. A fact important both to "man and horse," at all events, to the breeder of the latter.

Mr. Budd alludes to the opinion of Laennec that bronchitis is the ordinary cause of emphysema, the mucous secretion opposing the free exit of air—and to Louis, whose dissections and researches disproved this frequent antecedence of bronchitis. He adds—

"Laennec was right in supposing that dilatation of the air-cells is occasioned by an obstacle to the free escape of their contents; but he was wrong in believing this obstacle to exist generally in the bronchial tubes. Louis was correct in stating that emphysema often comes on without the previous occurrence of bronchitis; but he was, I believe, in error, when he ascribed dilatation of the air-cells to a cause different in its nature from that which produces dilatation of other organs. Dilatation of the air-cells, like dilatation of the chest, is a necessary consequence of want of elasticity of the lung. The powerful muscles of inspiration are continually acting to dilate the chest, and thence, by virtue of atmospheric pressure, the air-cells. This agency is not counteracted as it should be, by the natural elasticity of the lung; and the air-cells, as well as the cavity of the chest, are in consequence permanently dilated.

I have already shown that the other anatomical characters of emphysema, together with most of the symptoms of this disease, result from the same cause; and I am, therefore, led to consider the absence of elasticity * of the pulmonary tissue as the fundamental character and primary condition of emphysema of the lungs." 53.

Such is Dr. Budd's view. We may be permitted, however, to remark that no proof is offered in support of it. The emphysematous lung is less elastic than it should be, but is that elasticity the consequence of the emphysema, or the emphysema of it, or do they play into one another's hands?

Dr. Budd next passes to the subject of asthma.

This has been generally ascribed to constriction of the small bronchial tubes, from spasm of the circular fibres surrounding them. But Dr. Budd, with others, disputes the muscularity of the fibres in question. To satisfy himself, he performed, some experiments with the assistance of Mr. Busk.

A rabbit, between two and three months old, was killed by a smart blow behind the ears. As soon as its struggles were over, the trachea was taken out, and the anterior part of the cartilaginous rings removed by the scissors, in order that any motion produced by the transverse fibres at its posterior part might be more readily seen. When a portion of the trachea, thus prepared, was placed on a plate, not the slightest movement could be seen in it, nor could any be excited by the wires of a galvanic battery. One of the lungs was then removed, and placed on the plate, between two and three minutes after the struggles of the animal had ceased. The end of the bronchi in which the lung terminated, was obstructed by light froth. No motion could be observed in this froth, or in the lung, before or after the wires were applied to different points on the surface of the lung, neither could any motion be perceived, when the lung was cut into, and the extremities of the wires were placed near one of the bronchial tubes.

The abdomen was opened at the end of five minutes, when the muscular fibres of the stomach and intestines were seen to contract slowly, but very

---

* "Magendie ascribes the difficulty of breathing in emphysematous persons and in broken-winded horses, to want of elasticity of the lung, but he does not attribute the dilatation of the air-cells to the same cause: on the contrary, he says, 'par suite de la rupture d'un certain nombre de cellules, et de la dilatation d'u certain nombre d'autres, le tissue de l'organe a perdu de son elasticité, et il ne reagit plus avec une energie suffisante sur l'air qui a penetré dans son parenchyme.'" Leçons, t. i. p. 169.

distinctly, under the galvanic influence. At the end of ten minutes, these contractions were no longer perceptible; but vigorous contractions could still be excited in the heart, and in the muscles of the larynx.

Another rabbit, of the same age as the former, was killed in the same manner; one of the lungs was taken out as quickly as possible, and placed on the plate. Not the slightest movement could be observed in it, nor could any be excited by placing the wires of the battery at different points of its surface, or in contact with the bronchial tubes. The trachea was then removed, and treated as in the former experiment, and with the same result. The abdomen was next opened, the intestines were moving from peristaltic action. The muscles, both of the intestines and of the stomach, which was distended, contracted very distinctly when galvanism was applied. At the end of ten minutes from the death of the animal, these contractions were no longer discernible; but more than half an hour after, contractions could be excited in the heart, and in the muscles of the larynx.

Passing over some objectionable experiments of Varnier's, Dr. Budd refers to Wedemeyer's, one of which is thus described by Müller.

" Wedemeyer laid bare the trachea in a living dog, and freed it from cellular tissue for the space of two inches: he then cut out a portion in front, and irritated the posterior wall of the trachea mechanically and by galvanism, but could not produce the slightest contraction. Wedemeyer now opened the thorax quickly, and removed the lungs with their bronchi. He made several sections of the larger bronchi, but could discover no sign of contractility in them. On applying galvanism, however, to the smaller branches of about one line in diameter he thought he saw them undergo a distinct contraction, but it took place very slowly." 58.

Dr. Budd observes:—

" This experiment of Wedemeyer, as far as the trachea and larger bronchi are concerned, agrees with those that I have before related: and together they seem to establish that no contractions can be excited in those tubes by galvanic influence. This point, if admitted, affords an almost conclusive argument against the muscularity of the smaller tubes. For the transverse fibres in smaller tubes have the same arrangement as in the larger, and we cannot suppose them to be of different nature without admitting a break in the law of continuity. The resemblance of the transverse fibres of the bronchial tubes to the muscular fibres of organic life—the chief argument in favour of the muscularity of the former—is certainly more striking for the fibres in the larger than for those in the smaller bronchi. The contraction witnessed by Wedemeyer in tubes of a line in diameter, resulted in all probability from chemical changes, especially the coagulation of albumen, caused by the galvanic influence. Such changes were very manifest in my own experiments. The manner in which I performed the experiment, by placing the wires, not in contact with one of the small bronchial tubes, but at different points on the surface of the lung, affords a much more delicate test of the muscularity of the bronchi. If these were muscular, a great number of them would be excited at once when the wires were placed on the surface of the lung and the galvanic influence diffused through its mass: and their combined effect would be visible in movements of the surface of the lung, or of the froth obstructing the orifice of the terminal bronchus." 59.

Dr. Budd uses another argument. If, says he, the bronchial fibres are muscular, they must be of the involuntary kind. But the external muscles are voluntary. Then there may come a want of accord between the two. For we might will to breathe more quickly, and the bronchial muscles might not, on the instant, act so. We confess that this objection does not seem so conclusive to us as to its author. The muscles of deglutition are voluntary in the mouth, less under the control of the will in the pharynx, and removed from it in the œsophagus. Yet things go on pretty well, whether we eat fast or slow. Only suppose an association of action through the medium of the nerves, and the difficulty vanishes.

The following reasoning is, perhaps, more conclusive.

"The idea of spasm of the bronchi was suggested to Cullen, and has been generally adopted, from inability to explain in any other way the symptoms of asthma. A little consideration, however, is sufficient to show the improbable nature of this supposition. The large bronchial tubes, and, in man, those even of the fourth and fifth ramifications, cannot be closed by reason of the cartilaginous rings or plates, which wholly or partially surround them. Supposing, then, the circular fibres to be muscular, only very small bronchial tubes could be closed by their action; and the closing of a few of those tubes would only obstruct the passage of air to the small portions of lung to which they lead, and would not cause much difficulty of breathing. The spasm, to explain the symptoms of asthma, must be supposed to affect the small bronchial tubes in a considerable portion of the lungs; and as, in almost all cases of asthma, some shortness of breathing remains, in the intervals of the fits, we must admit, and, in fact, most physicians who have written on the subject have admitted, that some degree of spasm is permanent." 61.

The fact that diseases of the heart and great vessels, and that emphysema of the lungs are frequent causes of the "asthmatic dyspnœa," prevents, no doubt, in many cases, the necessity for resorting to the hypothesis of spasm. There still, however, says Dr. Budd, remain some cases, which at present we can only explain by supposing the dyspnœa to be nervous. It seems probable that the number of such cases will be still further diminished, and that many of those fits of asthma, which we are now forced to consider nervous, will be discovered to depend on some organic change which has as yet escaped our observation, perhaps on some morbid condition of the blood itself. If the asthma be really nervous, Dr. B. concludes that it depends on spasm or suspension of the normal action of the diaphragm and other muscles of inspiration.

### V. On a remarkable Effect upon the Human Gums produced by the Absorption of Lead. By Henry Burton, M.D., &c.

Dr. Burton has examined, since 1834, the mouths of patients admitted into his wards, who had been exposed to the action of lead in the course of their usual avocations; and of those also who had swallowed the acetate of lead medicinally. "The result," he adds, "of this investigation has proved highly interesting. It has led to the belief that a salivation in the ordinary sense of the word does not occur in one case out of thirty-six cases of lead colic, the number examined in my wards; nor in one case out of fourteen cases of pulmonary disease, which were treated by me with acetate of lead; but in the total number of fifty patients who were examined whilst under the influence of lead, a peculiar discolouration was observed on their gums, which I could not discern on the gums of several hundred patients, who were not under the influence of lead, and which I believe cannot be produced by any other internal remedy." The sign, therefore, becomes diagnostic of the presence of lead, and may obviate in many cases the infliction of lead colic.

The following is a sufficiently accurate account of the appearances. The edges of the gums attached to the necks of two or more teeth of either jaw, were distinctly bordered by a narrow leaden-blue line, about the one-twentieth part of an inch in width, whilst the substance of the gum apparently retained its ordinary colour and condition, so far as could be determined by comparing the gums of these patients with those of other patients of the same class in the hospital: there was no invariable tumefaction, softening or tenderness about them; neither was there any peculiar fætor in the breath, nor increased salivary discharge to be observed on any of the fifty patients; and on thirteen out of fourteen patients, who were treated in the hospital with acetate of lead, and carefully watched during its employment, the substance of the gums, the smell of the breath, as well as the quantity and colour of the saliva, preserved the same characters, after the appearance of the blue line, as they respectively possessed before the saturine preparation was administered; but on the fourteenth patient, who died from hæmoptysis, the gums, which were, previously to the use of lead, tumid and soft, became contracted and firm, after the blue line had appeared.

In no one instance, did Dr. Burton observe any thing at all similar to the bleeding tumefied gum of scorbutus. Nor was such a state of gum as is induced by mercurial salivation ever observed by him.

The discolouration is a very constant occurrence; it precedes all other unequivocal symptoms produced by lead, and is not equally exposed to the imputation urged against most medical data, of being fugitive and deceptive. For the discolouration is very permanent; it has endured through months and until death, and having been once observed may be afterwards easily recognised. On a few patients only had it entirely disappeared before they quitted the hospital; on others it had only partially vanished. In many it continued with little or no change; and on a few patients who died after the medicinal use of lead had affected their gums, the discolouration appeared more distinct a few hours after death, and before putrefaction could have began than during life. It cannot be confounded, when distinct, with the ordinary colour of the gums, during life; and after death any ambiguity which might have existed previously will be entirely removed by the strong contrast of colours disclosed on the gums of the dead body. The pathognomic value of the discolouration will bear a proportion to the regularity of its appearance under similar conditions; and in some cases, a little ambiguity may arise from the difficulty of discriminating between imperfectly defined colors; but this ambiguity will soon cease if the patient continues exposed to the action of fresh portions of lead; and in all cases the phenomenon will possess some importance if viewed in connexion with the ordinary symptoms of the presence of lead.

The sign in question, has enabled Dr. B. to detect the presence of lead in the system, when the patients themselves were not aware of it. Dr. Burton relates two rather interesting cases.

The *first* was that of a carpenter. He had never worked in lead, nor had he any suspicion of having been exposed to its influence; but he had experienced a severe illness about four years before his admission into the hospital, which had been followed by a partial paralysis of the fingers of his left hand. In other respects his health was restored, and continued good until a few weeks before he was placed under Dr. B.'s care; he then began to feel languid, and to experience a sense of weight about the limbs; his appetite failed, and subsequently he suffered a pain in the stomach, which extended upwards over both breasts to the shoulders and down the arms; the bowels had been constipated for a week previous to his admission, and during this interval vomiting had several times occurred. His nights had been passed without sleep; his pulse was 96, soft and regular, his skin warm, his countenance pale. In addition to these symptoms tremors were noticed in both hands when the patient extended his arms, and the gums were very distinctly marked with a leaden-blue border line. The combination of symptoms in this case was such as indicates lead colic and paralysis of the wrists; but in what manner lead was introduced into the system could not be ascertained.

The *second* patient was a cordwainer, who had, until his admission, resided in the country. The features of this man were sallow; he was spare, entirely free from paralysis of the voluntary muscles, but he had experienced for several years, at intervals, repeated attacks of colic, by which he had been confined to his bed seventeen times. During these attacks he had endured violent pains in the abdomen, frequent vomiting, and obstinate constipation, sleepless nights and loss of appetite. The gums of this patient were rather turgid, although not more so than nine-tenths of the gums of those patients who resort to hospitals; they were also very well marked with the peculiar blue line, but no other evidence of the patient having been exposed to the action of lead could be obtained.

Dr. Burton believes, and it is very probable, that however ignorant they might be of the *mode*, these patients were labouring under the influence of lead. Our author, indeed, ventures to express a strong notion that the unobserved introduction of lead into the human body is continually taking place, to a much greater extent than is usually imagined, and that it has often caused an ambiguous assemblage of morbid symptoms: for although the influence of lead on the system is readily detected when the symptoms are severe and follow each other in the expected order of succession, yet when they are mild or do not follow each other in the regular and stated order of succession, if the mind of the physician is not awake to their cause, or the cause cannot be ascertained, then the symptoms appear ambiguous, and they may be misinterpreted without exposing the physician to the imputation of unpardonable ignorance, or of culpable oversight. But he contends that, in abdominal diseases simulating lead colic, as well as other forms of disease about which any ambiguity exists, an inspection of the gums will decide the question, whether the symptoms were produced by lead. Thus, cases often occur in hospital practice in which the functions of the brain and cerebral nerves are paralysed by lead, and in which coma, vertigo, headache, amaurosis, and sometimes deafness, are the most evident effects; in other instances the patients complain of articular pains resembling those of chronic rheumatism, periostitis, and secondary syphilis. In many of these cases an inspection of the gums will assist in making a correct diagnosis.

He thinks that articular pains, proceeding from the action of lead, have been treated sometimes as those of chronic rheumatism, at others as those of secondary syphilis, often empirically. And he quotes a case from Andral —that of a painter, who never having experienced lead colic, suffered during four or five months severe pains in the membranes of the head, which had been at first regarded as rheumatism, and unsuccessfully treated by bleeding and vapour baths; but there being afterwards reason for believing the pains were produced by lead, the patient was treated for ordinary lead colic, and recovered.

"The next problem to be solved is, whether the phenomenon can be made available as a means of averting the infliction of lead colic in the treatment of disease with saturnine preparations. To give an incontestable solution of this problem would require a greater number of data than I have hitherto been able to collect; nevertheless, on referring to my ward-books of the last few years, there appear to have been about twenty-seven patients treated with acetate of lead and opium; and out of that number, there were twenty at least in whom no colic and no other material inconvenience was induced by the remedy, except constipation; in two or three cases the colic symptoms were very severe, but in these latter the hæmorrhage was profuse, and the dose only proportionately large. But with ordinary precautions, colic does not occur severely during the medicinal use of lead; and I have frequently persevered in the use of the salt, for some time after the blue line had appeared, without producing it, or only slightly." 66.

The time required to produce the blue line varies in general with the amount of the dose, but not always; and, *cæteris paribus*, large doses affect the gums sooner than small. Mr. Moyle of Chacewater produced the discolouration in twenty-four hours, by giving four doses of gr. v. each, every six hours; and Dr. B. thinks it very probable, that in cases of poisoning, from "the irritant effects of large doses of the soluble salts of lead," similar to those described by Dr. Christison in his very valuable "Treatise on Poisons," the discolouration would be obvious on the gums in five hours after swallowing the salts; although the time required in several cases under Dr. Burton's own care, was much longer, in which large medicinal doses of the acetate were given frequently in twenty-four hours.

Dr. B. suggests an examination of the gums in medico-legal investigations, and he also suggests to workmen the precautionary examination of them in their own persons.

### VI. A CASE OF DISEASE IN THE POSTERIOR COLUMNS OF THE SPINAL CORD. By EDWARD STANLEY, Esq. F.R.S. &c. &c.

Mr. Stanley is so well known as an accurate anatomist and pathologist, that any fact which he relates will carry with it the greatest possible weight. The case which forms the subject of his present paper, derives much of its value from the eminent character of its author.

*Case.*—Joseph Cosden, aged 44, was admitted into St. Bartholomew's Hospital on account of the loss of the power of motion in his lower limbs, of which he gave the following history: that it had not been preceded by any external injury, and had commenced about three years previously; that at first, and for some time, the impairment of motion was slight, but had afterwards progressively increased to the present period. In the investigation of the case on its admission, the patient was lifted into a chair; and when thus sitting, he did succeed, by a great effort, in raising his legs from the ground; but afterwards the inability of motion became complete through each lower limb in its entire extent. There was no discoverable impairment of sensation in any part of either limb: on scratching, pricking and pinching the skin, nowhere was any defect of feeling acknowledged by the patient. In the upper limbs there existed no defect either of motion or sensation. The general health was feeble. In the idea that the impairment of the lower limbs might in some degree depend on congestion in the vessels of the spinal cord, a few ounces of blood were taken, by cupping, from the loins; which reduced the pulse, and occasioned the feeling of extreme debility, but with no improvement in the limbs. Mercury was also administered to the extent of inducing moderate salivation, but with no benefit. The further symptoms were simply those of gradually increasing exhaustion of the vital powers, with inability to expel the urine or retain the fæces. Quinine, ammonia, wine, with the most nutritive diet, produced a slight and transient impression on the general health, but none on the condition of the lower limbs. At length he sank, about three months from the period of his admission into the hospital.

The case was supposed to be one of disease of the anterior columns of the spinal cord. It turned out to be the contrary.

*Dissection.*—The cord was found to be the only seat of disease, but that this disease was strictly limited to its posterior half or columns. About an ounce of serous fluid was found in the theca of the cord; in other respects the membranes were healthy. The substance of the cord through its posterior half or columns, and in its entire length, from the pons to its lower end, had undergone the following changes of colour and consistence: it was of a dark brown colour, extremely soft and tenacious. The substance of the cord, through its anterior half and entire length, exhibited its natural whiteness and firm consistence; and on making a longitudinal section of the cord through its centre, and in the antero-posterior direction, the boundary line between the healthy and diseased nervous matter was seen to be most exact: it was a straight and uninterrupted line from the pons to the lower end of the cord. The roots of the spinal nerves were unaltered. The brain was healthy. The mucous membrane of the bladder exhibited the characters of recent inflammation. The kidneys and other viscera were sound.

Mr. Stanley observes that the examination of the body, in this instance, was witnessed by many competent observers—that it proves the uncertainty of our knowledge of the functions of the anterior and posterior columns of the cord—and, finally, that we have no means of explaining the continuance of sensation and motion in the upper limbs, while the cervical portion of the cord exhibited such alteration of structure. If well-authenticated cases are on record, in which destruction of a portion of the cord throughout its entire thickness has not led to the abolition of sensation or motion below the seat of disease, that does not, unfortunately, assist in elucidating, however it may support such cases as the present.

### VII. On the Arrangement of the Intermediate Vessels on Surfaces secreting Pus; with a Note regarding the Vascularity of Articular Cartilage. By Robert Liston, &c. &c. &c.

Mr. Liston is well known by his friends to have devoted much time to the microscope, and to be very expert in the use of it. He is not likely to play with it, nor to allow what it discloses to be lost to surgery. We must all feel indebted to him for the following observations on a point of much importance—the mode in which granulations are formed and receive their vascular supply.

Mr. Liston remarks, that the granular deposit of lymph on surfaces exposed and unprotected by integument, is speedily supplied by blood-vessels, nerves, and absorbents, admits of no doubt, and is easily demonstrated by the examination and treatment of any healing ulcer. Mr. Liston describes the arrangement of the intermediate vessels on granulations, as they appear in the cysts of abscesses, and on open sores.

"It will appear, on careful examination, that the abscess is coated on the interior and free surface by a layer of lymph of greater or less thickness, as may be; generally, about one-tenth of an inch. This layer is first of all deposited in a fluid state, and consists of the liquor sanguinis, or fibrine in a state of solution, as separated from the blood. It is exuded in the form of minute transparent drops, which being spontaneously coagulable, gradually become milky and consistent. The granules appear first of all to become coagulated on the surface, and the interior of the drop, as it were, remains for a time fluid and transparent. A sort of minutely granular or tuberculated surface externally, cellular internally, is thus formed.

This layer, with which the purulent deposit is in immediate contact, by and by becomes more consistent, and acquires a yellowish white colour. It lies upon a highly vascular membrane, to which it adheres more or less intimately, according to the duration of the process. The vessels in this tissue are curiously interlaced, anastomosing freely with each other, so as to form a very fine and delicate net-work.

There seems to be in this lymph, from the first, an impulse, as it were, towards organization; and after a very short time it becomes permeated by minute blood-vessels, which admit our fine injections. The diameter of the vessels was most frequently $\frac{1}{2000}$th of an inch. The extreme sizes being $\frac{1}{4000}$th and $\frac{1}{1333}$rd; and the following intermediate measurements were obtained, viz., $\frac{1}{3000}$th, $\frac{1}{2000}$th, $\frac{1}{1660}$th, and $\frac{1}{1500}$th, of an inch.

These capillaries project into the new and adventitious membrane from that underneath it; often, in straight parallel lines. Their arrangement in the granules on the free surface is, however, distinctly looped and tortuous; and these loops communicate with each other." 87.

Which, asks Mr. Liston, is the "pyogenic membrane?" The deposit of lymph in the greater number of situations and circumstances precedes the secretion of pus; and when this layer becomes organized, and the vessels assume the curiously convoluted and looped arrangement shown above, there can be no doubt but that the office of secretion is performed there. The resemblance to the looping of vessels in healthy secreting surfaces, the skin, the mucous membranes, &c., must be obvious.

Mr. Liston further inquires—how these looped vessels are produced. It is not easy to imagine that they are mere elongations of the original capillaries of the part, which have been dilated and relaxed. The deposit, as already remarked, seems to have an internal impulse towards organization. Mr. Hunter suspected that new parts had the power of making vessels and red blood independently of the original circulation—a view supported by observations on cold-blooded animals.

In solutions of continuity, proceeds Mr. Liston, reparation takes place, as has been well known since the time of John Hunter, by the deposit of plastic matter; and this layer, as that distinguished pathologist has shown, is speedily supplied by blood-vessels. On a careful examination of a portion of injected ulcer, more particularly in a profile view of it, it will be found that the secreting vessels are arranged in a precisely similar manner to those in granular deposits of lymph. This might have been expected, and so might the slight difference in appearance. These vessels on exposed surfaces are disposed in exactly the same fashion; but they are also enormously and irregularly dilated—varicose in fact. This arises, no doubt, from want of support, or from unfavourable position. And in neglected ulcers the vessels of the granulations often burst. The dark colour of the sore, the bloody and gleety discharge, very soon show to the surgeon of experience in hospital practice whether or not the patient obeys the injunction to keep the limb elevated. Soon also, does he discover whether or not, any trick is attempted by ligature or otherwise, to interrupt the progress of cure.

The purulent secretion is probably transuded through the coats of the looped, tortuous, and dilated capillaries.

As regards cicatrix, it may be remarked, that the vessels speedily contract. They are arranged in a reticular fashion, but, after a time, the net-work is not nearly so full as in the surrounding skin.

Occasionally, an approach to the papillary arrangement seems to be attempted, as seen in good sections after successful injections.

Mr. Liston offers a few practical deductions.

" And first of all, the mischievous effects of squeezing together the sides of suppurating cavities may be noticed.

By this proceeding, adopted through a blind and thoughtless observance of the bad practice of others, the lymphatic coating is separated from its vascular base; the circulation of the part is unnecessarily excited; bloody and often putrid secretion is poured out; and the general health in consequence disturbed. If a sufficient opening is made in a dependent position, the accumulated secretion is rapidly enough discharged; and the walls of the cavity come together and coalesce through the natural elasticity and action of the parts.

As regards ulcers, the paramount advantage of an elevated position of the affected part must be sufficiently obvious. The rapid disappearance of congestive swelling, and of inflammation by an observance of this practice alone, in many cases, must make apparent the good effects of favouring the return of blood.

The larger veins, previously varicose and over distended, become collapsed, and almost disappear.

The same effect upon the varicose capillaries in the solution of continuity necessarily follows; the colour of the sore is speedily altered for the better, the painful feelings abate, and the nature of the discharge is ameliorated. Until this is the case, and as long as over-action, to any degree exists, soothing and relaxing applications are advantageous; exudation of lymph and plentiful secretion of pus are thus encouraged.

These are followed by mild astringents and stimulants, by which the dilated and weakened condition of the coats of the vessels may be supposed to be amended.

The discharge is thus moderated, and the granulations prevented in a manner, from becoming exuberant. The beneficial effects of uniform support can also be well understood." 92.

Mr. Liston concludes by alluding to the question whether cartilage is extravascular or not.

He has been enabled to demonstrate the existence of vessels, most undeniably, in the articular cartilage of several diseased joints, and presents a sketch of one portion. In this the vessels run straight, in parallel lines, from the injected membrane of the bone. Many of these are joined at their further extremity in the cartilage, thus forming long loops. The possibility of cartilage being acted upon, nourished, absorbed, and repaired, by its own vessels, must thus be admitted. In fact, in many of the specimens in Mr. Liston's possession, lymph is deposited on the surface of ulcerated cartilage, and injected vessels can be traced passing into this lymph. Mr. Liston concludes:—

"Under circumstances favourable for it, solutions of continuity in cartilage appear to be repaired, without however much reproduction of the tissue.

It would appear that ulcerative absortion of cartilage occurs in three forms:

First. In consequence of disease of synovial membrane, which becomes much swollen, and to which processes of adventitious tissue are superadded, the cartilage is removed where it is encroached and pressed upon. The prolongations of the membrane, in a highly injected state, as well described by Mr. Key, fit most accurately to every crevice of the breach of surface in the cartilage. At first there is no union of the surfaces, the membrane being merely accurately adapted and closely applied to the ulcerated surface. Frequently, however, as the disease advances, adhesions form betwixt the vessels of the synovial membrane, and those proceeding from the medullary web. An adhesion of considerable length is thus often formed betwixt the synovial surface, and the articulating end of the bone.

Second. Absorption of cartilage seems often to arise from swelling and intense vascularity of the tissue connecting it to the bone. This cellular tissue is scarcely demonstrable in the healthy condition of parts, any more than is the vascularity of the articular cartilage; but it becomes most remarkably developed in a state of disease. The cartilage is in consequence loosened and thinned; at first, apparently, by interstitial absorption. Then it becomes perforated, and an ulcer, of greater or less extent, with thin undermined edges, is presented. In consequence of disease of the interposed tissue, the cartilage is sometimes thinned, and ultimately detached in flakes; forming, in fact, sequestra of the tissue.

Third. Lastly, cartilage still firmly adherent to the subjacent bone, is permeated by vessels communicating with those of the bone, and ulceration proceeds from the free surface. The cartilage, very often previously swollen and softened, is gradually and irregularly thinned: the bone is exposed, and is finally acted upon also, by ulcerative absorption. The ulcerated surface is generally coated by a layer of organized lymph. More than one form of the ulcerative process may sometimes be observed in the same articulation." 95.

A valuable communication.

### VIII. Remarks on the Diagnosis of Foreign Bodies in the Larynx. By Cæsar H. Hawkins, Esq. Surgeon to St. George's Hospital.

Mr. Hawkins' observations are always of so practical a bearing as to render them equally interesting and instructive. The following quite support that character.

Mr. Hawkins commences by relating a case.

Nov. 18, he was asked to see Miss S. aged 12 years, who had been suddenly seized, while taking some soup about eight hours before, with violent vomiting, and suffocating cough, which lasted for a short time, and then left her with a noise in breathing, which was somewhat difficult, and with a sense of pain beneath the cricoid cartilage. She believed she had felt a piece of bone in her mouth at the time, and that she had swallowed it. About two hours after the accident an emetic had been administered by Mr. Davis, the assistant of a medical man in the neighbourhood, which had brought up some solid meat, and seemed to have a little relieved her.

When Mr. Hawkins saw her she was breathing with a croupy noise at each inspiration, but without much labour, and she complained of some pain and tenderness in the larynx, referred more particularly to the cricoid cartilage. She could swallow without any difficulty, and on examination with a pair of curved forceps, it was evident that there was nothing in the œsophagus at the seat of the pain. The finger, passed behind the epiglottis, felt nothing like a foreign body in that situation; her voice was natural, and there was no cough, nor had there been any since the accident, to which attention would otherwise have been drawn. The tongue was a little dirty, she was flushed and the eyes suffused, and the pulse quickened, and there was some anxiety of expression. She had been in good health before the accident, except that she had a slight cold the day before, with a sense of tightness across the epigastrium. The lungs appeared healthy, and there was no other apparent cause for the croupy noise and difficulty of respiration, except a good deal of fullness and redness of the tonsils and palate and fauces, which might extend to the larynx, but which might also have been the consequence of the vomiting occasioned by the accident and by the emetic.

Mr. H. had little doubt that a piece of bone had "gone the wrong way," but under the circumstances he thought it advisable to leave her till morning, administering some calomel and antimony, and applying a sinapism to the throat.

Next morning, there was neither distress in breathing, nor feverishness, but the noise in respiration was as constant as before, and was equally audible in expiration and in inspiration, and a little pain and tenderness remained below the cricoid cartilage. Both Mr. Hawkins, however, and Mr. Babington, who saw the case with him, concurred on the propriety of not delaying the operation.

"I therefore made the usual opening into the trachea, just below the thyroid gland, which was unattended with any hæmorrhage, and removed a small piece of two rings of the trachea, in the centre of the incision, which was made through three others also, and endeavoured to get the piece of bone thrown out by making the patient cough repeatedly, but without avail; feeling the foreign substance with the probe just above the opening, I then introduced a pair of forceps and extracted it, not without some little violence, from the manner in which it was fixed. It was a portion, as it seemed, of the spine, shewing the curved surface of the canal of a vertebra in a neck of mutton, nearly half an inch long, and a third of an inch wide, the outer surface being very rough and irregular, so as to account for its fixed position below the glottis." 100.

The breathing immediately became noiseless, and neither cough nor other unfavourable symptoms succeeded.

Mr. Hawkins makes some observations on the case, which, we think, should receive the serious attention of surgeons.

1. In by far the greater number of instances, a foreign body which has entered the windpipe continues to be moveable within the trachea. Mr. Hawkins quotes Mr. Ryland's account of the symptoms. " From laryngitis or croup," says Mr. R., " this accident may be distinguished by the absence of fever at first; by the very sudden manner in which the symptoms came on; by the *intermission* in the difficulty of breathing, which sometimes continues for an hour or two; by the *noise* occasionally heard when the foreign body is impelled against the vocal cords; by the *excessive violence* of the *cough* after this occurrence, and most *particularly* by the chief difficulty of breathing being during the time that the *expiratory* process is going on; whilst in laryngitis the chief difficulty is in the act of inspiration."

Mr. Hawkins observes on this:—

" No doubt this account is generally correct, when the intruding substance is *within the trachea*, even when it has been surrounded by tenaceous mucus, causing it, at the time of operation, to be adherent to the membrane of the tube, so as not to be immediately expelled, as in a case of Sir Charles Bell's at the Middlesex Hospital; or when it has adhered by some roughness, as in a case of a piece of the jaw of a mackarel, extracted by Pelletan. Yet it will be observed, that in the preceding case scarcely one of these symptoms corresponded with what really took place; the attack was indeed sudden, so as by itself to render the case scarcely doubtful; but there *was* a good deal of feverish excitement when I first saw the patient; there was *no intermission* whatever in the difficulty of breathing, and for the same reason *no noise* could be heard by the striking of the substance against the vocal cords; there was absolutely *no cough* whatever after the first few seconds; and instead of the noise in breathing being chiefly in *inspiration*, it was heard, on the day of the accident, only in *expiration*, and on the following day it was equally audible in *both portions* of the respiratory process." 102.

2. When a foreign body is moveable within the trachea, it has been frequently found to pass into the right bronchus; and some interesting cases of this kind have been published by Mr. Key, Dr. Houston, Mr. Liston, and others, who have shown the absence of the symptoms before enumerated, if it remains almost entirely in this situation, together with the new stethoscopic signs of its presence in the bronchus, viz., the freedom of the larynx from disease, and the occasional or permanent cessation of respiration in the lung of the affected side.

3. If the foreign body is actually fixed within the vocal cords, instant and sudden death has usually been the immediate result; whether it has been impacted in this situation at once, or has first moved freely within the trachea, and has been subsequently fixed in the glottis during a fit of coughing.

4. If it is fixed within the larynx in some other situation, as in the ventricles, without causing immediately fatal effects, a foreign body is yet generally said to occasion much distress and danger. ' It will produce,' it is said by Dr. Stokes, ' more or less *violent* and *incessant attacks* of *cough* and *dyspnœa*, in which the lungs are found, on auscultation, to be sound, and the larynx to be the seat of the constriction; the permanency of which, together with the history, will point out the nature of the case.' 'It may happen,' says Mr. Porter, 'that if the body be round and polished and small, it shall occasion no symptom of distress, except *the cough*, and the *difficulty* of *breathing*, and the patient may exist for a long time without the occurrence of those morbid actions which render the accident certainly fatal."

5. "But," says Mr. Hawkins, "that a foreign body should be fixed in the larynx *below* the glottis, and that the symptoms should be much modified by this position, does not appear to have been noticed by writers upon this subject; except that the cough, in cases of foreign bodies within the air-tube, arising from the direct irritation of the glottis, the absence of this symptom, it has been remarked, may be considered as a presumptive proof that the foreign body is fixed somewhere in the tube. Even this remark, however, requires some correction, since it must be recollected that the fatal effects upon the *lungs* may occasion cough, although without so much distress, as in the cough produced by direct irritation of the glottis. The part of the larynx immediately below the glottis is not enumerated by any writer, as one of the situations in which a fixed foreign body is to be looked for, although some distinctions have been attempted to be drawn of the symptoms likely to be occasioned by the different situations I have before alluded to, viz., the glottis itself, or one of the ventricles of the larynx, or the tracheal tube, or one of the bronchi." 105.

Mr. Hawkins has only been enabled to find two cases similar, though not precisely like his own. These he refers to, and concludes by remarking:—"It appears then that in three cases in which a foreign body was fixed in the situation of the cricoid cartilage below the glottis, the severe paroxysms of coughing, which are invariably looked for as evidence of the presence of a foreign body, (but which really belong essentially to its presence in other parts of the tube,) were entirely absent in two, and were mild in the third, so as to lead the surgeon to believe they could not arise from the entrance of the pebble, as the child asserted, and were afterwards entirely absent in the last month of her life;—that even the voice was unaffected in two of the cases, although hoarse in the third case;—but that in all three cases there was soreness and uneasiness in the part where the foreign body was fixed, a *noise* in inspiration or expiration, or in both, from the mechanical effect of the intruding substance, (mistaken indeed for croup in one of them,) and in all, the *patient asserted* that something had been *swallowed*.

Where such circumstances as these are present to guide the surgeon, I conceive he is imperatively called upon to operate without much delay, since out of the only three cases, with which I am acquainted, in which the foreign body has been thus lodged and fixed near the glottis, two were fatal; one within sixty hours, by the immediate effects upon the lungs, though without any other symptom than in my own case; the other at a later period, by the slower influence of inflammation; while in my patient a more fortunate result was met with, in consequence, it cannot be doubted, of the removal of the foreign body, at an early period, by an operation, which is seldom very difficult, except in very young children, and perhaps is never attended with any important risk."

### IX. History of a Case in which Tracheotomy was performed; with Observations. By Benjamin Travers, Jun. Esq.

We are glad to perceive that Mr. Travers, jun. is following in the steps of his able father. Similar zeal in the prosecution of surgical science will lead, we cannot doubt, to similar eminence, a prize of no mean value to the most ambitious man.

*Case.*—A robust girl, aged six years, the daughter of a farmer residing near Eye in Suffolk, whilst sitting upon some straw in the yard, was suddenly thrown backwards by a pig concealed beneath the heap. She was eating cherries at the moment, and was immediately seized with a violent fit of choking, and every symptom of impending suffocation. This condition lasted an hour, and then she fell asleep. The accident occurred about four o'clock in the afternoon of Friday, July 19th. She was seen an hour afterwards by Mr. Vincent, surgeon, of Rickinghall. Being awakened by slight cough, some emetic and purgative medicine was given, but no stone was detected in the matters rejected from the stomach and bowels.

She slept well during the ensuing night, but on the 20th, had some spasmodic pain in the chest. At seven a,m. of the 21st, there being dyspnœa and inflammatory symptoms, some blood was drawn and calomel and opium were given.

At four p. m. of the 22d, she had violent convulsive seizure with cough, small quick pulse, a livid surface, suffused eye, and every sign of threatened suffocation. The spasm subsided in two hours, when she was tranquil till the 23d, when she had another fit. Cough frequent and sonorous. On the 25th, there was another paroxysm after which there was no disorder except occasional cough. The child remained well till the 1st of August, when the spasmodic attack returned with great violence, lasting two hours. The seizure now recurred daily, varying as to degree and duration.

Mr. Travers first visited the child in company with Mr. Vincent on the evening of Wednesday the 7th instant. She had just recovered from a severe fit of cough, and was sleeping uneasily. When roused, her breathing was laboured and stridulous, pulse small and hurried, countenance anxious and suffused; there were frequent paroxysms of croupy cough, attended by great restlessness, and that peculiar grasping of the throat before noticed. The temperature was sustained. The attacks were now more frequent, and the consequent exhaustion more marked.

Mr. Travers lost no time in performing the operation. With a little difficulty, he succeeded in dividing vertically three rings of the trachea with the connecting membrane midway between the isthmus of the thyroid gland and the top of the sternum. By inclining the head forward, an oval aperture was produced, of sufficient extent to have permitted the escape of the stone, had it been free to move in the canal. The breathing became tranquil, and the cough ceased. Mr. Travers determined to ascertain whether the larynx was obstructed. He pushed a silver catheter fairly through the glottis, which he ascertained by passing his fore-finger over the base of the tongue, so as to touch the apex of the instrument in that situation.

The gums were made sore—towards the close of September the wound healed—in the beginning of October the child coughed incessantly, and had night sweats, with loss of strength and appetite. Mr. Vincent still suspected the presence of a stone, and on October 23d, his suspicion was verified by the ejection of a stone, together with a table-spoonful of pus, during a violent paroxysm of cough. From this time the cough never returned, and the general health was soon re-established.

Mr. Travers makes some very pertinent and useful observations on the case, but having already, in our notice of the preceding paper, devoted much space to the subject, we are constrained to pass them over with this simple commendation.

### X. Memoirs on some Principles of the Pathology of the Nervous System. By Marshall Hall, M.D. &c. &c.

Dr. Marshall Hall has done and is doing good service to physiology. There are few persons, we think, possessed of a more untiring zeal in the advancement of science than himself. To say less would be injustice.

This Second Memoir is on:—

### The Morbid Reflex and Retrograde Actions of the Spinal Marrow.

He first treats of—

*The Reflex Actions.*—Dr. Hall begins by observing that Haller, Bichat, and Müller distinctly state that the *vis nervosa* acts in *one* direction only, viz. that *from* trunk *to* branch, or *from* the nervous centres *towards* those parts of the muscular system placed in relation with them. But Dr. Hall observes, that it appeared to him that, when he had established that the reflex actions did not depend upon sensation and volition, but upon some other principle of the animal economy, the only known principle which remained, and which could be the probable agent in these actions, was the *vis nervosa.* He resolved, therefore, to institute a new series of experiments in order to determine the question, whether the *vis nervosa* were susceptible of other and unsuspected modes of action. These experiments consist in denuding and stimulating the lateral nerves in the decapitated turtle. It results from them, in reference to the *vis nervosa :*—

1. That it acts in *direct* lines *along* the spinal marrow, and *from* the trunks *to* the branches of the nerves, and *to* the muscles they supply,—according to the law laid down by Haller, Bichat, and Professor Müller.

2. That it acts in *reflex* directions *to* and *from* the spinal marrow ; that is, *from* peripheral, cutaneous, and mucous surfaces, *through* the spinal marrow, and *to* the co-ordinated muscles, according to a newly-discovered law ; and, as will be seen hereafter,

3. That it acts in a *retrograde* direction along the spinal marrow.

Now, under what circumstances, are reflex actions most apparent in the human frame? Dr. Hall can state that, in order that they may be very apparent, it is essential—

1. That the interference of *volition* should be removed ;

2. That the *vis nervosa* and the *vis muscularis* should be unimpaired, not to say augmented, and

3. That the *reflex nervous arcs* should be uninterrupted.

The interference of volition with some of the phenomena of the reflex function is obvious from some of the phenomena of sleep and of comatose and paralytic affections. Gently touch the palm of the hand of a sleeping child, it grasps the finger—do the same thing when the child wakes, it does not. Again, in cerebral paralysis, the reflex actions are most observed when the paralysis is most complete.

"The first effect of a violent experiment or accident seems to be to suspend the *vis nervosa,* the *vis muscularis,* or both. It is accordingly observed that *immediately* after the division of the spinal marrow, in an experiment, or *immediately* after injury sustained by the same organ in the human subject, by a fall or other accident, the reflex actions subsequently developed and manifested most clearly, are not observed.

The nervous and muscular powers are gradually restored from this suspension as the effect of shock, and, at a still more remote period, even acquire an anormal degree of intensity. The phenomena dependent on them are augmented proportionately. The same remark is still more true in regard to cases in which the *vis nervosa* is morbidly augmented by disease, as in tetanus, hydrophobia, certain affections of the spinal marrow, in the effects of strychnine, &c. In these latter cases the slightest cause of excitement is reflected with terrific energy upon the appropriate parts of the muscular system.

3. Lastly, it is essential that the reflex nervous arcs should be entire. It has been observed that in some cases of paraplegia the reflex actions are present, in others absent. A slight knowledge of the anatomy of the spinal column is sufficient to explain this apparent discrepancy. If the disease be seated within the *cervical* or *dorsal* vertebræ, the spinal marrow in this part is affected, but a portion *below* may remain free from the influence of the disease ; the reflex arc which involves this portion may, therefore, be entire, and the reflex actions will be observed. If, on the contrary, the disease be situated within the *lumbar* vertebræ, the cauda equina is affected, the centre of every reflex arc is excluded, and all the reflex actions will be absent." 126.

Dr. Hall gives a table of the nervous arcs through which reflex actions take place—a table which we insert.

ANATOMY OF THE TRUE SPINAL SYSTEM.

I. The Incident Motor Branches.

1. The Trifacial arising from—

   1. The Eye-lashes.

   2. The Alæ Nasi.

   3. The Nostrils.

   4. The Fauces.

   5. The Face.

2. The Pneumogastric, from—

   1. The Pharynx.

   2. The Larynx.

   3. The Bronchia.

   4. The Cardia, — Kidney, and Liver.

3. The Posterior Spinal, arising from—

   1. The General Surface.

   2. The Glans Penis or Clitoridis.

   3. The Anus.

   4. The Cervix Vesicæ.

   5. The Cervix Uteri.

   6. The Extremities.

II. The True Medulla Oblongata and Medulla Spinalis, the Centre of the System.

III. The Reflex, Motor Branches.

1. The Trochlearis } Oculi.

2. The Abducens } Oculi.

3. The minor portion of the Fifth.

4. The Facial, distributed to

   1. The Orbicularis.

   2. The Levator Alæ Nasi.

5. The Pneumogastric or its Accessory

   1. The Pharyngeal.

   2. The Œsophageal and Cardiac.

   3. The Laryngeal.

   4. The Bronchial, &c.

6. The Myo-glossal.

7. The Spinal, distributed to the

   1. Diaphragm, and to

   2. The Intercostal and } Muscles.

   3. The Abdominal } Muscles.

8. The Sacral, distributed to

   1. The Sphincters.

   2. The Expulsors, the Ejaculators, the Fallopian Tubes, Uterus, &c.

   3. The Extremities.

Dr. Hall next proceeds to make some observations on the diseases in which the reflex phenomena are observed, and to give a series of cases in illustration.

1. *Diseases of the Head.*—In the coma of apoplexy, of epilepsy, and of hydrocephalus, we observe, according to the *degree* of the affection, the diminution of the cerebral, and of the cerebral and true spinal functions. The test is supplied by the eyelids. In the slighter forms of coma, the eyelids are frequently but partially closed, yet they close perfectly on touching the eye-lashes; in the severer forms of this affection, not only the cerebrum, but the medulla oblongata, has its powers impaired, and the eyelids do not close, although touched.

2. *Of Hemiplegia.*—The reflex actions are not less observed in cases of hemiplegia than in cases of paraplegia; but as they are, in general, more obvious the more complete the paralysis, and as the paralysis of hemiplegia is, in general, less complete than that of paraplegia, they have been less observed in the former affections.

Dr. Hall gives several instances of this. One only need be cited.

*Case.*—"Mr. F., aged about fifty-five, was seized, three months ago, with apoplectic symptoms, which left pretty complete hemiplegia. At first there was a little stertor and a little dysphagia; but these symptoms ceased with the apoplectic state, the former at once, the latter a little more tardily. There was also slight eneuresis for several days. On tickling the sole of the foot, or pinching the skin, or pulling a hair of the leg, and on applying a spoon just taken out of hot or cold water, there were distinct sudden movements of the leg. The same thing occurred in regard to the arm, but in a less marked degree. On first applying galvanism, the paralytic arm was least affected: the effect I suppose, of the shock of the disease; afterwards the paralytic arm was most moved, as in other similar cases. On the same principle, the effect of emotion, as laughter, was at the first more observed on the healthy than on the paralytic side of the face; more remotely, the equilibrium of the countenance, under the influence of laughter, was restored, or nearly so. At this time, the arm, and especially the hand, are paralysed to voluntary motion, but readily agitated by emotion, and sudden or energetic respiratory efforts, and constantly contracted as by a spring, the arm towards the trunk of the body, the fingers towards the palm of the hand; and, lastly, more agitated by the influence of galvanism than the unaffected limb. The voluntary power of the arm is much less restored than than that of the leg, in which the phenomena just enumerated are, comparatively, absent." 137.

3. *Of Paraplegia.*—Dr. Hall details also many cases of the existence of the reflex actions in paraplegia. One will be a sample.

*Case.*—A girl, about 15 years of age, who was a patient of Mr. Crosse, at the Norfolk and Norwich Hospital, a few years since, was affected with angular curvature of the spine, producing insensibility and paralysis of the lower extremities. On tickling the *soles of her feet*, which as an experiment was often done, the legs were immediately slightly retracted, although the patient said she felt nothing; it was further remarked that on touching the *other parts of the feet* or *the legs*, in the same manner, no effect was produced.

4. *Tetanus; Hydrophobia; Effects of Strychnine.*—Dr. Hall observes that—" as in cerebral paralysis we have augmented irritability of the muscular fibre, or of the *vis insita*, in tetanus and hydrophobia we have the *vis nervosa* morbidly augmented, but in an infinitely greater degree.

The slightest external stimulus is sufficient to excite reflex actions in their most terrific forms.

What is remarkable is, that it is precisely the functions of the orifices and sphincters, of the ingestors and egestors, which are most affected in these formidable diseases; and, most of all, the larynx, the pharynx, the organs of respiration, and the rectum.

The remarks which have been made relative to the condition of the reflex function in tetanus and hydrophobia, apply equally to that artificial tetanus induced by strychnine. In a report of La Charité, of Berlin, drawn up by Dr. Köhler, it is observed that, " in some individuals, the sensibility to external impressions, under the influence of strychnine, was so great, that they broke out into an almost uncontrollable fit of laughter on being touched with the finger."

5. *Undue Excitability.*—Instead of paraplegia, and the other forms of paralysis, arising from disease of the spinal marrow, we have occasionally undue excitability. Dr. Hall remarks that it is still a question how far the spinal marrow is primarily or organically affected in these cases, which he thinks quite distinct from those of common paraplegia.

*Case.*—In one case there were movements of the fingers somewhat like those seen in chorea, whilst the muscles of the legs were spasmodically contracted; the patient was as incapacitated for muscular exertion as in paraplegia. The skin was so susceptible to impressions in certain parts of the surface, that the patient was affected with a sort of general emprosthotonic spasm, with a slight sob whenever the bedclothes, for instance, were drawn over his chest, and still more especially when the penis was accidentally touched in a similar manner. Similar effects were observed on applying the pure potassa to establish an issue along the spine. The legs were drawn upwards whenever the sole of the foot first touched the cold floor on rising in the morning.

6. *Peculiar Dysphagia.*—" From an undue excito-motory action, the pharynx seizes some solid portion of what is attempted to be swallowed, and this is afterwards returned by a peculiar effort, for which I know of no designation but that of a forcible hawking. A pill, though taken with a large draught of water, is arrested at the upper part of the pharynx. A little of the core of apple, or of the gristle of meat, is seized and retained in the same manner, the rest being duly swallowed. Sometimes large portions of food are thus retained. When the pharynx is thus occupied by a portion of food, it is necessary to remove it either by swallowing some fluid, or by the effort just described. It may not be without interest to add, that I am myself affected with this singular kind of dysphagia."

7. *Morbid Action of the Rectum, and Bladder, and of the Sphincters.*—There is, says Dr. Hall, a peculiar affection of the rectum and bladder in some nervous affections, of which the following experiment affords both the type and illustration : if, in a turtle, after the removal of the tail and the posterior extremities, with the rectum, and, of course, with a portion of the spinal marrow, water be forced into the intestine by means of Reads's syringe, both the cloaca and the bladder are fully distended before any part of the fluid escapes through the sphincter; which it then does only on the use of much force, and *by jerks*. If, when the cloaca is distended, the integuments *over it* are stimulated, the water is propelled to a considerable distance.

When the rectum or bladder is distended, the patient feels a sudden call, and the action of the expulsors is so energetic, or the power of the sphincters is so diminished, that unless the call can be promptly obeyed the fæces or urine escape.

In tenesmus and strangury, the sphincter of the rectum and of the bladder is excited to undue contraction respectively. A ligature applied to hæmorrhoids not uncommonly induces spasmodic action of the cervix vesicæ and retention of urine. In one case, calculus of the bulb of the urethra induced spasmodic stricture of the sphincter ani. All examples of morbid reflex action.

8. *Localization of Action of certain Remedies.*—Strychnine acts upon the glottis, cantharides on the neck of the bladder, aloes on the rectum, the secale cornutum on the uterus,—all organs specially under the influence of the excito-motory power and reflex function of the spinal marrow.

9. *Excitants of the Reflex Action.*—On the expulsion of the fœtus, and by the contact of the atmospheric air with the minute distributions of the incident nerves of the excito-motory system, that the functions of ingestion and egestion first commence.

It is, doubtless, from the impression of the atmospheric air on the trifacial and spinal nerves, distributed upon the surface of the face and body, that the first inspiration is excited.

The influence of cold water dashed on the face, and the influence of the diffused contact of the cold bath, in exciting sudden sobbing acts of inspiration, are well known.

The same contact of cold air which excites the first inspiration, also excites the first acts of expulsion of the fæces and urine. This effect is also seen in the late periods of existence. The cold bath induces the same effect. The uterus is well known to be very susceptible of the influence of cold too—the catamenia being checked, or uterine contraction excited by it. It is interesting, says Dr. Hall, to observe the influence of the same cause in disease. None are more remarkable instances of this than the phenomena observed in the coma of epilepsy and apoplexy. The medulla oblongata being compressed, together with the other contents of the cranium, the influence of dashing cold water on the face may be absolutely null: on taking off that pressure by blood-letting, the susceptibility to the influence is again restored: it becomes a measure, even, of the diminished compression.

There are other influences of cold, which must not be passed over unnoticed. Free exposure of the face to the cold breeze is the most effectual remedy in sickness, and affords manifest relief in asthma.

As to other excitants of the reflex functions, we need only call to mind the simplest facts. The nipple or the finger, introduced between the lips of the new-born, or even the anencephalous, fœtus, immediately excites the act of sucking: the mere introduction of the enema pipe into the rectum of an infant, equally excites the action of the rectum. The irritation of a few grains of common salt, applied to the border of the sphincter ani, will induce the premature expulsion of an egg in a common fowl.*

Food is the natural excitor of the pharynx, œsophagus, and cardia—the fæces and urine of the expulsors about the rectum and of the bladder.

Some internal textures are, however, capable of transmitting the influence of excitants. I have seen the limbs of the decapitated turtle moved energetically on dividing internal tissues; and I have known spasmodic affections induced by disease of similar internal tissues.

"It still remains for us to trace the influence of excitants of this function in some more hidden cases. It is almost certain that the gall-ducts, the ureters, and other excretory canals, are endowed both with incident and excitant, and with reflex and motor, nerves. The passage of a biliary or urinary calculus *excites* vomiting: exposure to cold, a loaded intestine, certain passions, and in infants mere dentition will, on the other hand, arrest the flow of bile and induce icterus.

The influence of the excitants of this system of actions, considered as *remedies*, is little known. One of the most interesting examples of this kind is that of the application of cold to the face and to the general surface, in some cases of suspended animation. As a remedy in the cases of the still-born fœtus and of drowning, the sudden contact of cold water is most important. I have already alluded to the use and influence of the cold water douche in cases of hæmorrhage from inaction of the uterus." 157.

10. *Retrograde Action in Spinal Disease.*—This subject is considered by our author to be involved in obscurity. It has been observed, he says, that an irritation of the middle part of the spinal marrow, below the origin of the brachial plexus, induces in some decapitated animals, and especially the cold-blooded and the very young of the warm-blooded, distinct movements of the anterior extremities.

"I removed the head of a young turtle: on pinching and galvanizing the lower extremity of the medulla oblongata, there was an excited act of inspiration. The same event occurred on stimulating the nostril, the intra-maxillary or palatine fringes, and the internal part of the larynx.

I then laid bare the middle portion of the spinal marrow by removing part of the shell. On pinching or galvanizing this, *both* the *anterior* and *posterior* fins were moved.

I took a frog, separated the head, and divided the spinal marrow low in the back: I then stimulated the lower end of the upper portion of the spinal marrow with the forceps; the anterior extremities moved in the most remarkable manner:—they were gently raised, without being affected with the *twitchings* seen in the inferior extremities when the upper part of the lower half of the divided spinal marrow was stimulated.

I was next anxious to perform these experiments on an animal of warm blood. I chose for this purpose a rabbit of six days old.

---

* The same effect is said to have been produced by the secale cornutum, in an experiment performed by M. Velpeau.

I first removed the head. I then stimulated the lower end of the divided medulla. There was an immediate act of *gasping*; I then divided the spine in the back, and stimulated the lower end of this middle portion of the spinal marrow; the anterior extremities were immediately moved." 159.

Dr. Hall, in reference to the question, whether retrograde actions of the spinal marrow take place in disease, cites three cases which, however, do not seem to us very satisfactory. We therefore pass them by.

Referring to these cases, and to the subject that they are intended to illustrate, Dr. Hall observes:—

"It is obvious that the question agitated in this place is one of great moment in the diagnosis of diseases of the spine; for if there be in disease or accident, retrograde influences of the spinal marrow, we must not always conclude that the disease or injury is situated *above* the origin of the nerves affected. It is equally obvious that the whole subject needs new and accurate observation.

I trust that one advantage will arise from the brief remarks which have been made in this communication, viz., that in every case of cerebral or spinal disease, and disease of the nerves in their course, the condition of the reflex actions, and of the retrograde influences of the spinal marrow and nerves, will henceforth be carefully examined. The first of these subjects has already made great progress; the second has scarcely been touched upon in medical writings. I will venture to suggest that cases of caries of the vertebræ appear to afford the most probable example of diseases *limited* to a given region of the spinal marrow, and therefore the best for the latter kind of inquiry. They afford examples of *irritation* before morbid processes have induced *disorganization*. The questions to be considered are two. 1. Is there paralysis? 2. Is there spasmodic action, in parts receiving their nerves from portions of the spinal marrow *above* the seat of the disease." 163.

And Dr. Hall concludes by submitting to the Society a certain number of propositions—inferences from the facts stated or observations made.

1. It is proved by the series of facts which have been observed in the human subject, that the excito-motory reflex actions are independent of sensation and volition, however they may be accompanied by sensation, or influenced by volition, in the perfect animal.

2. It is proved as a consequence, that the reflex actions are dependent on another principle of the nervous system; and it is proved by a series of experiments, that this principle is the *vis nervosa* of Haller, acting according to a new reflex law.

3. The phenomena of the excito-motory reflex actions are obvious in cases of paralysis, in proportion as that paralysis is more complete; they are therefore, more observable in paraplegia, than in hemiplegia, in general, but in each of these according to their intensity; they are therefore not only independent of sensation and volition, but inversely as these, frequently disappearing as these return.

4. In accidents, as in experiments, the excited reflex actions are not immediately observed, but are manifested only after the lapse of certain intervals of time; it is plain therefore, that the first influence of shock, is to diminish the excito-motory power; and this may remain until the patient falls a prey to the accident; as in the case noticed in Dr. W. Budd's paper.

5. It is observed that at a subsequent period, in more favourable cases, the excito-motory power is not only restored to its normal condition, but morbidly augmented.

6. This is especially observed in certain diseases, as tetanus, the effects of strychnine, &c.

7. The reflex arcs of the nervous system will be imperfect in cases of disease or injury of the lumbar or other regions, as in the case noticed in Dr. W. Budd's paper, and the reflex actions will consequently be absent; a fact which affords, in its turn, an important source of diagnosis, as to the seat of the disease.

8. In certain cerebral affections attended by coma, the presence or absence of reflex actions, in the eyelids especially, gives us an index of the degree of severity of the disease.

9. Certain diseases, as hydrophobia, epilepsy, hysteria, and certain remedies, as strychnine, cantharides, &c., not only induce augmented excitability, but manifest their effects precisely upon the organs which are, physiologically, under the influence and dominion of the excito-motory power.

10. There are new forms of disease of the true spinal functions, not hitherto described, such as the dysphagia, the peculiar action of the rectum, &c., which have been briefly noticed.

11. Certain parts, as the sides of the thorax, the soles of the feet, &c., are more susceptible of the excitement in question than others.

12. Dr. W. Budd has very justly observed, that in many cases of violent reflex, and even convulsive actions, there was no sense of fatigue, and little emaciation of the muscles. In fact, fatigue is a cerebral state, and cannot be expected to occur in the cases in which the reflex actions are most observed; and emaciation is most obvious in spinal paralysis, in which the reflex arcs being interrupted, the reflex actions are also precluded from taking place. Fatigue *is felt* severely after violent attacks of epilepsy and other spasmodic diseases, in which the cerebral functions are afterwards restored.

### MEMOIRS ON SOME PRINCIPLES OF PATHOLOGY IN THE NERVOUS SYSTEM. By MARSHALL HALL, M.D.

### MEMOIR III.—ON THE DISTINCT INFLUENCE OF VOLITION, OF EMOTION, AND OF THE VIS NERVOSA.

Dr. Hall observes that there are *three* causes or principles of muscular motion in the animal economy, besides the motor contractile power in the nervo-muscular fibre itself; viz., *volition, emotion,* and the direct and reflex actions of the *vis nervosa.* He first illustrates that of *volition,* but it does not appear necessary to follow him there. He next passes to that of *emotion.* He dwells on its frequency in man, its force, and its effects in inducing and complicating diseases of the nervous system.

In healthy circumstances, he says, the influence of emotion is diminished or counteracted by that of volition. But not only does emotion remain in connexion with the hemiplegic limb when sensation and volition are severed from it, but that emotion exerts its influence precisely upon those muscular organs which are under the influence of the *vis nervosa,* or excito-motory power: viz., the orifices and sphincters, the agents of ingestion and egestion; and as the *vis nervosa* acts *directly* upon certain internal organs, as well as

*reflectively* upon those just mentioned, we find the heart, the intestinal canal, the organs of secretion, &c., especially under the influence of emotion.

"Sensation and volition thus are seated in the cerebrum and its prolongations; emotion in the true spinal and the ganglionic systems. It is this distinct view of the subject to which I wish to draw the attention of the physiologist and pathologist." 173.

"Another form," says Dr. Hall, "of muscular action, if not of emotion, is that seen in the muscular system in general, and designated *tone;* the effect, I believe, of the constant agency of the *vis nervosa.* Far less obvious during the healthy condition of the system, it is made very manifest in certain circumstances of disease, and on the first cessation of the animal functions in death. When the influences of volition are withdrawn in hemiplegia, the hand and arm become much and permanently contracted. The influence of the same power is observed immediately after death, in the phenomenon termed cadaveric rigidity." 174.

With Dr. Hall's great ingenuity and penetration, we cannot help suspecting a tendency to rapid generalization. For example, *cadaveric stiffening* is at once decided to be *tone,* although there are many strong arguments, to say the least, against it. And the history of the doctrine of the nervous system must teach circumspection, when we come to reason on such principles as emotion. But this *en passant.*

Dr. Hall proceeds to pass in review the various diseases of the nervous system calculated to illustrate the questions before him.

1. *Of the Diseases of the Cerebral System.*—Dr. Hall reverts to hemiplegia, which dissects and severs, as it were, the cerebral from the true spinal system, volition from emotion and the *vis nervosa.*

After several observations, Dr. Hall concludes that the effect of hemiplegia is to paralyse the power of volition on the opposite side of the body, whilst the influence of emotion on this side remains. The seat, the source of these, must therefore be different. Those of the former are higher in the cerebrum, those of the latter lower down,—below the disease, probably in the medulla oblongata. Volition acts along fibres which decussate and affect the opposite side of the frame; emotion, like inspiration, has probably its course along another set of fibres, which do *not* decussate.

We may conclude, then, that hemiplegia severs the different motor powers from each other, and demonstrates their individual and separate existence: the influence of volition is cut off; that of emotion is occasionally, that of the *vis nervosa* constantly, energetic. Paralysis in regard to volition; agitation on occasions of emotion; tonic contraction from the constant action of the *vis nervosa;* such are the facts presented to our observation and consideration.

2. *Of the Diseases of the True Spinal System.*—Tetanus may be taken as the purest example of disease of the true spinal system. Whilst it spares the cerebrum, and with it the intellectual functions, it affects all those organs and actions of ingestion and of egestion, and, in a word, of the excito-motory system, which are untouched by hemiplegia. Deglutition, respiration, defæcation, are variously impeded.

Traumatic tetanus, proceeds Dr. Hall, being a series of morbid reflex actions, affords the type of affections of the system of incident and reflex nerves, and of their combiner, the true spinal marrow. The morbid influence is also retrograde as well as reflex. In disease originating in the spinal centre, the effect is usually less general, because retrograde ; but it is not less marked because more limited.

Still more limited, in its effects, is disease seated in the reflex or muscular nerves. Such disease is seen in the cases of spasmodic tic and torticollis. Spasmodic tic frequently arises from the influence of exposure to cold ; the first effect is generally paralysis ; the second, tonic or clonic spasm.

Passing over some cases we may cite Dr. Hall's conclusions : namely—that the seat of volition is the cerebrum, and that its action is along the fibres which *decussate* in the medulla oblongata, and that the seat of emotion is below that of volition, and that it acts along fibres which probably do *not* decussate. In these respects the effects of emotion resemble those of respiration, as seen in yawning, and this function is known to act in a direct manner, from the medulla oblongata not decussating. The same remark, and for the same reasons, may be made in regard to the tonic action of the *vis nervosa.*

Volition has an object, an aim. Emotion and the *vis nervosa*, however subdued to certain laws impressed by the Creator, and destined to special purposes, are aimless on the part of the individual, nay, frequently *opposed* to his volition.

According to the views of M. Flourens, and according to the emphatic expression of Professor Müller, volition acts upon the fibres of the medulla oblongata, as the finger upon the keys of a harpsichord. So do emotion and the *vis nervosa.* Where then is the difference of the effect produced ? These agents act upon different instruments !—Volition along the intra-vertebral *chord* of cerebral *nerves ;* emotion, and the *vis nervosa*, upon the fibres of the *true* spinal *marrow.*

These memoirs are valuable to the last degree.

### On the Presence of Sulphur in Cystic Oxyde, and an Account of a Cystic Oxyde Calculus, in the Museum of University College, London. By Henry Bence Jones, B.A. &c.

Mr. Jones is a young gentleman who has devoted some attention to organic chemistry, and is likely to excel in it. The object of his present paper is to corroborate a statement which was first announced by M. Baudrimont in France, and, subsequently, confirmed by M. Thaulow, in Germany,—*that* cystic oxyde calculi contain sulphur in considerable quantity.

Mr. Jones identified a cystic oxyde calculus, of considerable size, in the museum of University College, London. Passing over a very scientific account of it, for which we must refer our readers to the paper itself, we may state what was made out by Mr. Jones in relation to the sulphur.

The mode, he informs us, recommended by Liebig for obtaining pure cystic oxyde was followed in examining the calculus with regard to the presence of sulphur. Of ten grains of the calculus, treated with ammonia, about nine grains were dissolved ; which is the proportion of cystic oxyde ; the remainder being animal matter and phosphate of lime. On the spontaneous evaporation of the ammoniacal solution, crystals were formed, of which five grains were treated with nitric acid free from sulphuric; violent action ensued, after the cessation of which, some crystals of pure nitrate of potash were dropped into the solution, and the whole, after being evaporated to dryness, was gently ignited in a platinum crucible. The white fused mass resulting was alkaline to test paper, and its solution neutralized with nitric acid, gave on the addition of nitrate of baryta an abundant white precipitate, insoluble in the last named acid. This precipitate, collected on a filter, washed, dried, and ignited, weighed 6·94 grains, corresponding to ·9576 of sulphur, or above 19 per cent. This is considerably less than the amount given by M. Thaulow, but from the smallness of the quantity operated upon, from its having been examined as crystallized from an ammoniacal solution, and from other sources of error, the result must by no means be considered as throwing any doubt on the correctness of Thaulow's analysis, while it confirms the existence of a large quantity of sulphur in cystic oxyde. Whether the sulphur be variable in quantity, or be sometimes altogether wanting, future observation alone can determine.

We have completed a pretty close account of the first half of the Society's volume. In our next number we shall return to, and conclude it, and we think our readers will agree with us in our high estimate of its value.

